# Diagnosis of Multidrug-Resistant Pathogens of Pneumonia

**DOI:** 10.3390/diagnostics11122287

**Published:** 2021-12-07

**Authors:** Maroun M. Sfeir

**Affiliations:** Department of Pathology and Laboratory Medicine, University of Connecticut Health Center, Farmington, CT 06030, USA; sfeir@uchc.edu

**Keywords:** pneumonia, antimicrobial resistance, multidrug resistant bacteria, phenotypic tests, molecular assays

## Abstract

Hospital-acquired pneumonia and ventilator-associated pneumonia that are caused by multidrug resistant (MDR) pathogens represent a common and severe problem with increased mortality. Accurate diagnosis is essential to initiate appropriate antimicrobial therapy promptly while simultaneously avoiding antibiotic overuse and subsequent antibiotic resistance. Here, we discuss the main conventional phenotypic diagnostic tests and the advanced molecular tests that are currently available to diagnose the primary MDR pathogens and the resistance genes causing pneumonia.

## 1. Background

Hospital-acquired, or nosocomial pneumonia (HAP), is pneumonia that develops 48 h or longer after hospital admission. Ventilator-associated pneumonia is an infection that develops 48 h or more after mechanical ventilation that is given through endotracheal intubation or tracheostomy [1]. Healthcare-associated pneumonia (HCAP) refers to pneumonia that is acquired in outpatients who have had exposure to health care facilities such as nursing homes, hemodialysis centers, or a recent hospitalization within the past three months [2]. Patients with HCAP were believed to be at an increased risk for infection with multidrug resistant (MDR) pathogens; however, more recent studies have revealed that the risk of disease with MDR was dependent on specific host risk factors rather than on various aspects of health care exposure [3]. Thus, the HCAP category was intentionally excluded from the 2016 ATS/IDSA guidelines to avoid inappropriate broad-spectrum antibiotic use [1]. Similarly, the International European Respiratory Society/European Society of Intensive Care Medicine/European Society of Clinical Microbiology and Infectious Diseases/Latin American Thoracic Association guidelines for managing HAP and VAP omitted HCAP as a category of pneumonia [4].

Antimicrobial-resistant bacteria are important causes for HAP and VAP [1]. Based on the standard terminology that was developed by the United States Centers for Disease Control and Prevention (CDC) and the European Centre for Disease Prevention and Control, MDR was described as acquired non-susceptibility to at least one agent in three or more antimicrobial classes: extensively drug-resistant (XDR) was defined as non-susceptibility to at least one agent in all but two or fewer antimicrobial types and pan-drug-resistant (PDR) was defined as non-susceptibility to all agents in all antimicrobial categories. These definitions are applicable if bacterial isolates are tested against all, or nearly all, of the antimicrobial agents within the antimicrobial classes without selective reporting and suppression of results [5].

The drug resistance in pneumonia (DRIP) score depends on both major (two points each) and minor (one point each) risk factors [6]. Major risk factors include previous antibiotic use, tube feeding, residence in a long-term care facility, and infection by a drug-resistant pathogen within the past year [6]. Minor risk factors include recent hospitalization within the previous 60 days, poor functional status, gastric acid suppression, chronic pulmonary disease, wound care, and methicillin-resistant *Staphylococcus aureus* (MRSA) colonization within the past year [6].

## 2. Epidemiology

In 2019, lower respiratory infections were ranked as the fourth leading cause of death and represented the world’s most deadly infectious disease based on the World Health Organization [7]. The 2017 National Vital Statistics Reports listed pneumonia as a major cause of death in the United States, accounting for 49,157 total deaths in 2017 [8]. Pneumonia is the most common cause of hospital admissions through the emergency department [9]. Pneumonia and post-pneumonia care costs represent a significant healthcare burden with acute and long-term attributable expenditures exceeding $43,692 [10].

HAP and VAP are often caused by multidrug resistant pathogens and represent a significant challenge [11,12]. HAP and VAP are also associated with higher morbidity and mortality rates than other types of pneumonia [13,14]. A multi-center, retrospective, observational study identified a 72.5% rate of initial antibiotic treatment failure in patients with healthcare- and ventilator-associated pneumonia [12]. A multidrug resistant organism was isolated in 52.4% of the patients [12]. Multivariate analysis showed that the presence of an MDR pathogen was statistically associated with a higher initial antibiotic treatment failure (odds ratio [OR] of 3.39, 95% confidence interval, 1.41–8.16; *p* = 0.007) [12].

## 3. Microbiological Diagnosis

Appropriate antimicrobial therapy is critical to optimize treatment outcomes, especially in pneumonia with septic shock. When a patient presents with a respiratory infection, it is imperative to understand the causative agents and the antibiotic susceptibility profile. The American Thoracic Society (ATS)/Infectious Diseases Society of America (IDSA) guidelines do not routinely recommend microbiological testing for outpatients with community-acquired pneumonia [15]. However, the ATS/IDSA recommends obtaining microbiological tests, including blood cultures, sputum Gram stain and culture, and rapid nasal PCR or culture for methicillin-resistant *Staphylococcus aureus* (MRSA) for hospitalized patients with severe respiratory tract infections to the general medical ward or the intensive care unit due to the concern for multidrug resistant pathogens [15].

The current standard diagnostic procedures include cultures of the infective organisms followed by strain identification and antibiotic susceptibility testing. More rapid approaches for diagnosing infections and resistance that do not rely on cultivation include polymerase chain reaction (PCR)-based methods (PCR assays, microarrays, and targeted sequencing efforts). Many of these tests still require further optimization and approval before becoming reliable diagnostic assays at the point of care.

### 3.1. Conventional Phenotypic Tests

Although the value of obtaining blood cultures for all hospitalized patients with community-acquired pneumonia is debated, two sets of blood cultures that have been obtained before empiric antibiotic administration in patients that have been hospitalized with severe pneumonia may enhance diagnostic yield [15,16]. Most experts agree that the lower respiratory tract should be sampled for culture, and peripheral blood cultures should be obtained for ventilator-associated pneumonia [1,17]. However, one should consider the yield of blood culture, the risk of isolating contaminants, and costs. A retrospective study by Zhang et al. showed that routine blood cultures have a meager yield and utility in community-acquired or healthcare-associated pneumonia [16]. On the other hand, a multi-center, observational, prospective study found a 14.6% rate of bacteremia in patients that were admitted with HAP or VAP [17]. Patients with bacteremia and HAP or VAP had a significant longer stay in the intensive care units compared with non-bacteremic patients with HAP or VAP (28.5 ± 30.6 vs. 20.5 ± 17.1 days, respectively; *p* = 0.03) and had a higher risk for ICU mortality (OR = 2.01, 95% CI = 1.22 to 3.55, *p* = 0.008) [17]. The 2017 European guidelines recommend using quantitative cultures for invasive lower respiratory sampling (e.g., mini-bronchoalveolar lavage, bronchoscopic BAL, or protected specimen brush). On the other hand, the Infectious Diseases Society of America/The American Thoracic Society states a preference for noninvasive sampling with semi-quantitative cultures for VAP diagnosis that is based upon evidence that demonstrates no difference in mortality or length of stay when compared to the invasive approach [1]. There is no evidence that using quantitative cultures of respiratory secretions result in lower mortality or a reduction in ICU stay and length of time on mechanical ventilation than qualitative cultures in patients with VAP [18].

In most cases, conventional microbiological testing is time-consuming and labor-intensive. The likelihood of false-negative bacterial culture results increase with concomitant antibiotic administration [19]. Moreover, in one study, one-third of episodes of VAP that were caused by *P. aeruginosa* were associated with negative direct staining [20]. Consequently, in most cases, negative direct staining requires initial broad-spectrum antibiotics until culture results are returned.

### 3.2. Molecular Testing

Molecular assays that detect multiple respiratory pathogens, including bacteria and viruses from a single respiratory tract sample, are being increasingly used. These assays are rapid and less labor-intensive than conventional methods. In addition to organism identification, the new multiplex amplification assays offer the advantage of detecting resistance genes. The significant issues of the new molecular methods are the cost and the competition in the market. In addition, positive results do not rule out coinfection with non-panel organisms or resistance genes. Thus, it is essential to interpret the results of the molecular panels based on the local epidemiology and resistance levels. While the use of multiplex panels increases the likelihood of detecting a microorganism in a respiratory tract sample, the predictive value of these results is not clear [21]. For example, the presence of a viral pathogen does not rule out the possibility of bacterial coinfection. Similarly, some viral and bacterial pathogens can colonize the airways and their detection does not definitively indicate infection. Moreover, the pretest probability of a given pathogen or disease prevalence influences the positive- and negative-predictive values of these assays and is, therefore, essential to diagnostic decision making [21]. Finally, not all respiratory molecular assays improve outcomes or save costs [21,22,23]. In sum, while these assays are promising, the true impact of multiplex molecular assays on patient outcomes is yet to be substantiated by data. The Biofire FilmArray Pneumonia Plus Panel (FilmArray^®^; BioFire Diagnostics, Salt Lake City, UT, USA) is US Food and Drug Administration (FDA)-cleared for lower respiratory tract specimens (sputum and bronchoalveolar lavage fluid). It is a comprehensive pneumonia test for 27 common pathogens and 7 genetic markers of antibiotic resistance, including *CTX-M*, carbapenemases (*KPC, NDM, Oxa48-like, VIM*, and *IMP), mecA/mecC* and *mec* element (*SCCmec*), and right extremity junction (*MREJ*) with a turnaround time of about an hour [24]. The sensitivity and specificity for pathogen identification are 96.2% and 98.3% for BAL, and 96.3% and 97.2% for sputum specimens, and for detecting antibiotic resistance, 100% and 98.5%, respectively [24]. Diatherix target enriched multiplex PCR (TEM-PCR) (TEMPCR™; Diatherix Laboratories, Inc.; Huntsville, AL) is another respiratory target-enriched multiplex panel that covers nine viruses and fifteen bacterial pathogens (*Acinetobacter baumannii, Bordetella pertussis, Chlamydophila pneumoniae, H. influenza, H. influenzae* (type B), *Klebsiella pneumoniae, Legionella pneumophilae*, *Moraxella catarrhalis, S. aureus*, MRSA, *Panton-Valentine leukocidin* gene, *Mycoplasma pneumoniae, Neisseria meningitides, Pseudomonas aeruginosa*, and *Streptococcus pneumoniae*) with a rapid turnaround time of 8 to 12 h from receipt of the specimen from the upper and lower respiratory tract [25]. This latter has not been FDA-approved yet. The Unyvero hospitalized pneumonia multiplex PCR panel (Curetis AG, Holzgerlingen, Germany) detects 21 bacteria including, *S. aureus*, most of the *Enterobacterales*, *P. aeruginosa*, *S. maltophilia*, or *A. baumanii* complex, well as 21 antibiotic resistance genes such as *CTX-M* subgroup 1 only, *SHV, TEM, KPC, IMP, NDM, VIM, OXA-23, OXA-24, OXA-48*, *OXA-58, mecA,* and *mec-C* genes [26].

Respiratory syndromic multiplex PCR panel on a nanofluidics platform detects common bacterial pathogens, e.g., *S. pneumoniae*, *H. influenzae*, *Klebsiella pneumoniae*, *S. aureus*, *P. aeruginosa*, *E. aerogenes*, and *E. cloacae*, as well as the detection of protein markers of resistance (*Van A*, *Van B*, *erm B*, *erm C*, *SHV*, *KPC*, *mef A*, *mec A*, *tet B*, *tet M*, *dfrA1*, *dfrA5*, *sul1*, and *sul2*) (Health Track Rx, Denton, TX, USA, https://www.contractlaboratory.com/labclass/directories/laboratories.cfm?Health-Track-Rx-Inc&i=2D9FBF47ECE72577B2681D3ED27A8979, accessed on 1 November 2021).

Clinical metagenomics that are based on whole-genome sequencing of clinical samples, could improve the diagnosis of HAP; however, many obstacles remain to be overcome, namely the turnaround time, the quantification of pathogens, the choice of antibiotic resistance determinants, the inference of the antimicrobial susceptibility testing from metagenomic data, and the linkage between antibiotic resistance genes and their host [27].

Broad-range bacterial PCR of a highly variable fragment of the 16S ribosomal RNA that is followed by massive parallel sequencing that is performed directly on fresh or frozen specimens, including respiratory samples and pleural fluid or formalin-fixed paraffin-embedded inviable tissue, could be helpful for the detection of pathogens if conventional culture remains negative due to pretreatment with antibiotics or infections with fastidious organisms.

Whole-genome sequencing determines the complete DNA sequence of a pathogen’s genome at one time, including resistance genes, multi-locus sequence type for identifying major lineages, and for species verification and phylogenetic clustering within clonal types to detect possible transmission. Due to the upfront cost of the sequencing instrument, data storage requirements, and data interpretation expertise, whole-genome sequencing is not widely available in clinical laboratories. Consequently, shipping specimens to a central or reference laboratory that performs the sequencing remains a challenge. The turnaround time remains long for the diagnosis of severe infection. As with the methods other molecular testing (e.g., PCR assays), sequencing does not differentiate between colonization and infection [28].

Nanopore metagenomics is a novel and promising technique that enables rapid clinical diagnosis within six hours of sample receipt of bacterial lower respiratory infection sample. This enables the identification of antibiotic resistance genes with a sensitivity and specificity of 96.6% and 41.7% for pathogen detection as compared with the conventional culture methods [29]. This study evaluated 40 respiratory samples by nanopore metagenomics; *S. pneumoniae* was missed in one of six culture-positive patients [29]. The clinical correlation remains essential in interpreting multiple pathogens detected by this method.

## 4. Microbial Etiology

There have been many multidrug resistant pathogens that have been implicated in respiratory tract infections. Here, we present a list of critical etiological agents of pneumonia.

### 4.1. Methicillin-Resistant Staphylococcus aureus (MRSA)

The WHO lists MRSA as one of the high-priority pathogens for the need for new antibiotics [11] and MRSA is one of the serious threats, based on the 2019 Antimicrobial Resistance Threats Report [7]. According to data that was reported to the National Healthcare Safety Network (NHSN) in the US from 2011 to 2014, *S. aureus* is the most common cause of VAP (24.7%) [30]. Both the *S. aureus* and *mecA* genes are part of most respiratory PCR panels, such as the FilmArray Pneumonia Plus Panel, Respiratory syndromic multiplex PCR panel, and the Unyvero hospitalized pneumonia multiplex PCR panel.

Small colony variants of *S. aureus* strains that are isolated in patients with cystic fibrosis, cause chronic staphylococcal infections that require long-term antibiotic therapy [31]. Small colony variants of *S. aureus* often need 48 to 72 h to grow on Petri dishes as pinpoint (approximately one-tenth the size of normal *S. aureus* morphotype) colonies with limited or non-pigmentation, reduced or non-hemolysis, and non-reactive critical biochemical tests [31]. Many SCV strains typically grow, although often not before 72 h of incubation, as pinpoint colonies or “fried-egg” colonies with elevated creamy colony centers [31]. 

These variants are often overgrown, overlooked, or misidentified. Mostly, they are difficult to detect [31]. For instance, they grow on the chromogenic agar media that was tested, but they do not exhibit the colony-specific color for the wild phenotype of *S. aureus*. They are frequently mannitol salt agar negative [31]. Thus, inoculation of blood agar plates should supplement the use of chromogenic or selective plates. Similarly, antimicrobial susceptibility testing is challenging due to the slow growth [31]. Essentially, longer incubation times must be applied before undertaking susceptibility testing [31].

### 4.2. Antibiotic-Resistant Streptococcus pneumoniae

Antibiotic resistant *S. pneumoniae* is one of the CDC’s serious threats and WHO pathogen with medium priority for antibiotic need [7,11]. According to the CDC, more than 30% of *S. pneumoniae* isolates are resistant to one or more antibiotics [11]. Pneumococcal infections in patients who recently used antibiotics are more likely to become resistant to antibiotics [8]. *S. pneumoniae* represents the most common etiology of bacterial pneumonia and pneumonia deaths worldwide [32]. MDR invasive *S. pneumoniae* infections have decreased in the US since pneumococcal conjugate vaccines were introduced [11,31]. Antibiotic resistance is rising among *S. pneumoniae* isolates across Europe, and susceptibility to macrolides, penicillins, and cephalosporins can no longer be predicted [33]. In China, a multi-center study showed that 46.1% of invasive clinical *S. pneumoniae* isolates were multidrug resistant [34].

*S. pneumoniae* is part of most, if not all, commercial respiratory panels. However, none of the commercial respiratory panels predicts the antimicrobial susceptibility of *S. pneumoniae*. The CDC *Streptococcus* Laboratory predicts phenotypic susceptibility results for a range of antibiotics using whole-genome sequencing data and machine learning algorithms [35]. For instance, a penicillin binding protein type sequence of *S. pneumoniae* isolates can predict resistance to β-lactam antibiotics, including penicillin [35].

### 4.3. Third-Generation Cephalosporin-Resistant Enterobacterales

According to the urgency of the need for new antibiotics, third-generation cephalosporin-resistant *Enterobacterales* are one of the CDC’s serious threats and critical WHO categories [7,11]. The third-generation cephalosporin-resistant *Enterobacterales* include the extended-spectrum β-lactamases (ESBL), which are plasmid-encoded, and the *AmpC*-type β-lactamases, which are now encoded by plasmid as well as chromosomal genes [36]. The most common ESBL type is *CTX-M*, followed by *TEM* and *SHV* β-lactamases [36]. According to the CLSI, testing isolates that demonstrate reduced susceptibility to cephalosporins for ESBL using Broth microdilution or disk diffusion clavulanate inhibition test is optional [37]. It is no longer necessary to change cephalosporins or penicillins from susceptibility to resistance because CLSI has updated cephalosporins breakpoints [37]. On the other hand, if laboratories have not implemented the current cephalosporin and aztreonam breakpoints, a ≥ 5 mm increase in zone diameter should be observed for cephalosporin tested in combination with clavulanate vs. the zone diameter of the cephalosporin that was tested alone [37]. A greater than or equal to three-fold concentration decrease in a minimal inhibitory concentration (MIC) was observed for cephalosporin when tested in combination with clavulanate vs. the MIC of the cephalosporin when tested alone [37]. Some respiratory panels can detect ESBL target genes (e.g., *CTX-M* detected by Biofire FilmArray Pneumonia Plus Panel, *TEM*, or *SHV* detected by the respiratory syndromic multiplex PCR panel on a nanofluidics platform). The incidence of ESBL infection increased by 53.3% (from 37.55 to 57.12 cases per 10,000 hospitalizations), a rise that was driven by an increase in community-onset cases based on a cohort of 890 US hospitals from 2012–2017 [38]. 

The Unyvero hospitalized pneumonia multiplex PCR panel detects the three ESBL target genes. However, ESBL testing remains useful for infection control and public health purposes [37]. The coexistence of multiple β-lactamases (an ESBL with an *AmpC* β-lactamase) can complicate the phenotypic identification of the β-lactamases. ESBL-producing *Enterobacterales* from respiratory samples may be detected on selective chromogenic media, e.g., CHROMagar ESBL, chromID ESBL, or Brilliance ESBL [39].

Unlike inducible chromosomal *AmpC* β-lactamase, the detection of plasmid-mediated *AmpC* β-lactamases are essential for infection control purposes. However, plasmid-mediated *AmpC* β-lactamases remain undetected in most clinical laboratories [40]. 

Phenotypic methods, e.g., *AmpC* disk test, boronic acid-based test, or cefoxitin disk approximation test, cannot distinguish between plasmid-mediated and chromosomal *AmpC* β-lactamase [40]. Thus, they are used to detect isolates without an inducible chromosomal *AmpC* gene, such as *K. pneumoniae*, and *Salmonella enterica* serovar Typhimurium [40,41]. The multiplex *AmpC* PCR test detects plasmid-mediated *AmpC* β-lactamases *MOX, LAT, DHA*, *ACC, ACT*, and *FOX* accurately (Perez-Perez & Hanson, 2002) [41].

### 4.4. Carbapenem-Resistant Enterobacterales

Carbapenem-resistant *Enterobacterales* (CRE) are listed among the CDC’s urgent threats and are a critical priority pathogen for developing new antibiotics according to WHO [7,11]. Hospitalized patients with CRE infections are challenging to treat and are associated with increased mortality rates of up to 50% [11,42]. Carbapenemase-producing CRE is a subset of CRE representing approximately 30% of CRE and is primarily responsible for the rapid spread of CRE [42]. Carbapenemase genes are carried on mobile genetic elements that are easily shared between bacteria, leading to the quick spread of resistance [42]. They can be diagnosed phenotypically by the modified carbapenem inactivation method and categorized phenotypically into metallo-β-lactamases or serine-based carbapenemases by the EDTA-enhanced carbapenem inactivation method (eCIM) [43,44]. A significant limitation for mCIM with or without eCIM is the requirement for overnight incubation. 

Rapid phenotypic tests for carbapenemase, e.g., CarbaNP, which detects changes in pH values of an in vitro hydrolysis of imipenem by a bacterial lysate using the indicator phenol red, may not consistently determine OXA-48 like carbapenemases [37].

The immunochromatographic complex lateral flow assays, i.e., NG-test CARBA-5 (NG Biotech) and RESIST-5 O.O.K.N.V. (Coris BioConcept) can also be used for the detection of the most common carbapenemases among *Enterobacterales* directly from bacterial colonies growing on Petri dishes in less than 30 min [45,46]. NG-test CARBA-5 is used for the phenotypic detection and differentiation of five common carbapenemases (*KPC, NDM, VIM, IMP*, and *OXA-48*-like) with the sensitivity and specificity of 100% and 100%, respectively [45]. RESIST-5 O.O.K.N.V. independently identifies *OXA-48*-like, *KPC, OXA-163*-like, *NDM*, and *VIM* (sensitivity and specificity of 99.4 and 100%, respectively), while IMP K-SeT identifies 18 IMP variants with the sensitivity and specificity of 100 and 100%, respectively [45].

Nucleic acid amplification tests (e.g., Xpert^®^ Carba-R, Cepheid^®^) or microarray tests detecting *KPC, OXA-48*-like *VIM, IMP* or *NDM* out of a colony in less than an hour determine the presence and the type of carbapenemase if the specific carbapenemase gene is targeted [37].

### 4.5. Multidrug Resistant Pseudomonas aeruginosa

According to the NHSN data report, *P. aeruginosa* is the second most common cause of VAP (16%) [30]; it is the most common Gram-negative causing VAP [30]. Along with carbapenem-resistant *Enterobacterales* and carbapenem-resistant *Acinetobacter* spp., carbapenem-resistant *P. aeruginosa* are considered the top critical priority pathogens in the WHO publication: Prioritization of pathogens to guide discovery, research, and development of novel antimicrobial agents for MDR [7]. Carbapenem-resistant *P. aeruginosa* is expected, with a population-weighted mean in Europe of 16.5% in 2019 [47]. In 2017, MDR *P. aeruginosa* induced approximately 32,600 infections among inpatients and 2700 estimated deaths in US healthcare facilities [11]. Carbapenem resistance in *P. aeruginosa* isolates entails several combinations of decreased production of the *OprD* porin channel for imipenem entry, *AmpC* β-lactamase overproduction, and the activation of efflux pumps such as *MexAB-OprM* and others [48,49]. An estimate of 2 to 3% of *P. aeruginosa* carries a carbapenemase enzyme that breaks down and inactivates antibiotics, including carbapenems [11]. Carbapenemase production can be tested phenotypically by the mCIM and CarbaNP [37,43]. While *S. aureus* is the most common pathogen that is isolated from respiratory specimens from young children with cystic fibrosis, *P. aeruginosa* is the most common in adults with cystic fibrosis [50]. *P. aeruginosa* can be reliably detected by conventional identification systems. It is identified by the majority of the respiratory panels that are available in the market that are FDA-approved and Communite-Europeenne (CE) marked (Table 1). CHROMagar^TM^ Pseudomonas (RambaCHROM *Pseudomonas*) is a selective isolation medium for *P. aeruginosa* (which forms blue colonies) from clinical respiratory specimens. 

The NG-Test Carba 5v1 (NG Biotech) would detect 89.4% of carbapenemase-producing *Pseudomonas* spp. missing some of the *IMP* variants [51]. The new version, NG-Test Carba 5v2 (NG Biotech) has an improved overall sensitivity of 97.4%, detecting all of the *IMP* variants without impairing the detection of the other four carbapenemases [52].

### 4.6. Carbapenem-Resistant Acinetobacter *spp*.

Carbapenem-resistant *Acinetobacter* spp. is one of the CDC’s urgent threats [11]. According to the WHO list, it is among the critical agents that desperately require novel antibiotics [7]. A prospective observational study in nine European countries showed that the *A. baumannii* complex was the most dominant cause of nosocomial pneumonia in ICU patients in Greece and Turkey [53]. Carbapenem-resistant *A. baumanii* complex often possesses plasmid-encoded *OXA* β-lactamases, commonly *OXA-23*, *OXA-40*, *OXA-51*, and *OXA-58* [54]. A retrospective matched cohort at two hospitals in Baltimore, MD, identified a significant association between patients with MDR *Acinetobacter* infection and prolonged hospitalization and ICU length of stay compared with susceptible *Acinetobacter* infection and uninfected patients [55].

CHROMagar^TM^ Acinetobacter can be used on nasal specimens to differentiate MDR *Acinetobacter* spp. for non-MDR *Acinetobacter* spp. and other Gram-negative bacteria that are inhibited on the medium (CHROMagar^TM^, Paris, France). MDR *Acinetobacter* spp. appear as red colonies after incubation in aerobic conditions at 35–37 °C for 18–24 h.

The immunochromatographic OXA-23 K-SeT^®^ assay (Coris BioConcept, Gembloux, Belgium) is a Conformite Europeenne (CE)-marked in vitro diagnostic test that is able to detect *OXA-23*-like carbapenemases-producing *Acinetobacter* spp. in cultured bacterial isolates rapidly [56]. However, this assay cannot screen other *OXA*-like variants reported in carbapenem-resistant *Acinetobacter* spp. i.e., *OXA-58*-like and *OXA-40*-like [56]. The immunochromatographic NG-Test Carba 5 (NG-Biotech) would detect only 12.9% of carbapenem-resistant *Acinetobacter* spp., mainly the metallo-β-lactamase producing ones [51].

### 4.7. Stenotrophomonas maltophilia

*S. maltophilia* is a nonfermenting MDR Gram-negative bacteria with several intrinsic or acquired antimicrobial resistant mechanisms [57,58]. It is uniformly resistant to carbapenems due to ubiquitous metallo-β-lactamase production [57]; the presence of an aminoglycoside acetyl-transferase and the temperature-dependent changes in the outer membrane lipopolysaccharide structure confer resistance to aminoglycosides [59,60]. It may colonize the respiratory tract and cause severe respiratory infections, especially in immunocompromised patients [61]. *S. maltophilia* is associated with wet surfaces and aqueous solutions. It has been reported to contaminate nebulizers in cystic fibrosis patients [62]. However, standard culture media, including blood and MacConkey agars, can grow *S. maltophilia*, VIA medium containing vancomycin, imipenem, and amphotericin B, improved *S. maltophilia* colony count that were recovered from cystic fibrosis patient sputum samples [63]. Standard biochemical tests, commercially available automated identification systems, and MALDI-TOF can accurately identify *S. maltophilia* [61]. *S. maltophilia* is included in most of the respiratory multiplex panels (Table 1).

### 4.8. Burkholderia cepacia Complex

*B. cepacia* complex (Bcc) is an emerging threat, especially in patients with cystic fibrosis, chronic lung disease, or chronic granulomatous disease [58,64,65]. It is intrinsically resistant to polymyxins and aminoglycosides and often acquires resistance to many other classes of antibiotics [64]. Microbiological identification of Bcc remains challenging for different reasons. First, Bcc does not grow on traditional culture media that are used in clinical microbiology laboratories [64]. For Bcc, respiratory cultures should be inoculated into selective isolation media, preferably on *Burkholderia cepacia* selective agar, or alternatively on oxidation-fermentation polymyxin bacitracin lactose agar or *Pseudomonas cepacia* agar [64]. Bcc may take two to three days to grow on those agars and often smell like dirt [64]. Second, conventional phenotypic testing that uses manual and automated systems may not be optimal for identifying Bcc to the species and even to the genus level [64,66]. MALDI-ToF MS, to some extent, and *recA* sequencing of a growing colony on a culture media are reliable for identifying Bcc at the species level [64,66]. Yet, Bcc is not part of a respiratory panel.

### 4.9. Drug-Resistant Tuberculosis

Drug-resistant tuberculosis is one of the CDC’s serious threats [11]. MDR *Mycobacterium tuberculosis* complex is resistant to isoniazid and rifampin, the two most potent tuberculosis drugs [67,68]. According to WHO, MDR tuberculosis constitutes 5% of the global tuberculosis cases [68]. However, less than 5% of patients with MDR tuberculosis are presently being diagnosed due to laboratory testing constraints [68]. Extensively drug-resistant (XDR) tuberculosis is resistant to the most anti-tuberculous drugs, including isoniazid and rifampin, plus any fluoroquinolone and at least one of the injectable second-line aminoglycosides (amikacin, kanamycin, or capreomycin) [67]. On average, XDR tuberculosis represents 9% of patients with MDR tuberculosis [69]. XDR tuberculosis remains scarce, with 63 cases that were reported between 1993 and 2011 in the US [67]. Microbiological testing of active *M. tuberculosis* complex respiratory infection includes acid-fast bacilli smear and mycobacterial culture of respiratory specimens, and molecular tests [67]. Conventional mycobacterial culture remains the gold standard test for detecting *M. tuberculosis* complex infection; it is the most sensitive tool, detecting as few as 10 mycobacteria/mL [69]. The sensitivity and specificity of sputum culture are about 80 and 98%, respectively [67,70]. Culture techniques use liquid media (faster growth, including the Mycobacteria Growth Indicator Tube [MGIT] which is FDA approved, and Microscopic Observation Drug Susceptibility (MODS) assay, not FDA approved) and solid media (Lowenstein-Jensen, Middlebrook 7H10 or 7H11) [69]. Nucleic acid amplification tests, e.g., the Xpert MTB/RIF assay and the Amplified MTD test, are FDA approved for respiratory samples from patients with suspected pulmonary tuberculosis [67]. The former is approved for respiratory specimens from patients with suspected tuberculosis who have been on less than seven days of treatment. It does detect tuberculosis but not drug resistance [67,69]. The Xpert MTB/RIF assay, which detects tuberculosis and rifampin resistance, is approved for only induced or expectorated sputum from untreated patients or patients on fewer than three days of therapy [67,69].

The conventional (phenotypic) susceptibility testing for *M. tuberculosis* complex uses an indirect proportion method to 12 anti-tuberculous drugs at 35 °C on Middlebrook 7H10 agar [67]. The test requires one month to complete. Molecular beacons are nucleic acid amplification tests to detect mutations in *rpoB*, *katG*, and *inhA* promoter region genes that are associated with rifampin, high-level and low-level isoniazid resistance, respectively, in one to two days [67]. Molecular Beacons test can be directly applied to clinical specimens or cultures. Compared with culture-based drug-susceptibility testing, molecular beacons showed 96 to 97% correlation [67]. Several commercial line probe assays that are available in different countries have demonstrated excellent agreement with conventional anti-tuberculous susceptibility testing methods, as well as high sensitivity and specificity to detect rifampicin and isoniazid resistance [68]. For example, the Genotype MTBDRplus assay (Hain Lifescience, GmbH, Germany) detects *rpoB*, *katG*, and *inhA* genes of both rifampicin and isoniazid resistance on isolates from solid and liquid culture as well as directly on smear-positive pulmonary specimens, whereas the INNO-LiPA Rif.TB (Innogenetics, Zwijndrecht, Belgium) detects the *rpoB* gene conferring rifampicin resistance on *M. tuberculosis* complex isolates grown on solid culture only [68]. Many microarray assays were developed that detect mutations conferring resistance to rifampin, isoniazid, ethambutol, streptomycin, ofloxacin, kanamycin, amikacin, and capreomycin simultaneously in six to seven hours [71].

## 5. Conclusions

In conclusion, MDR pathogens causing respiratory tract infections are complex and require complex diagnostic tools. They are a common and severe problem with an increased mortality risk [1]. The prompt identification of MDR pathogens is essential for decreasing the time to optimal definitive antimicrobial therapy. The current respiratory molecular panels are not available for all MDR bacteria and lack sensitivity for others. Not all molecular assays have shown decreased healthcare costs or improved clinical outcomes #2 [21,22,23]. Additionally, the need for clinical correlation and the expertise of the interpretation of results remains instrumental with the advance in molecular diagnostic testing. Therefore, further studies are needed to develop rapid, accurate, and cost-effective diagnostic tests to detect MDR pathogens and resistance genes.

## Figures and Tables

**Table 1:** Multiplex assays used for detection of multidrug-resistant pathogens causing pneumonia.

Test (Manufacturer)	Specimen Type	Microorganism Targets	Antibiotic Resistance Marker Targets	Turnaround Time	Regulatory Status	References
FilmArray^®^ Pneumonia Identification Panel (BioFire)	Respiratory secretions: sputum, endotracheal aspirate, BAL, mini-BAL	Bacteria (Semiquantitative identification) *Acinetobacter calcoaceticus-baumannii* complex *Enterobacter cloacae* complex *Escherichia coli* *Haemophilus influenzae* *Klebsiella aerogenes* *Klebsiella oxytoca* *Klebsiella pneumoniae* group *Moraxella catarrhalis* *Proteus* spp. *Pseudomonas aeruginosa* *Serratia marcescens* *Staphylococcus aureus* *Streptococcus agalactiae* *Streptococcus pneumoniae* *Streptococcus pyogenes* Viruses Atypical bacteria (qualitative identification)	Methicillin resistance: *mecA/C* and *MREJ* Carbapenemases: *KPC* *NDM* *Oxa-48*-like *VIM* *IMP* ESBL: *CTX-M*	1 h	US. FDA approved CE-IVD	[24]
Unyvero^®^ hospitalized pneumonia multiplex PCR panel (Curetis AG)	Endotracheal aspirate, BAL, mini-BAL	*Acinetobacter* spp. *Chlamydia pneumonia* *Citrobacter freundii* *Escherichia coli* *Enterobacter cloacae complex* *Haemophilus influenza* *Klebsiella oxytoca* *Klebsiella pneumoniae* *Klebsiella variicola* *Legionella pneumophila* *Moraxella catarrhalis* *Morganella morganii* *Mycoplasma pneumoniae* *Pneumocystis jirovecii* *Proteus* spp. *Pseudomonas aeruginosa* *Serratia marcescens* *S. aureus* *Stenotrophomonas maltophilia* *Streptococcus pneumoniae* Viruses	ESBL: *CTX-M* (subgroup 1 only) Carbapenemase: *KPC* *NDM* *OXA-23* *OXA-24* *OXA-48* *OXA-58* *VIM* Methicillin resistance: *mecA* Penicillin resistance: *TEM*	4 to 5 h	US. FDA approved CE-IVD	[26]
Diatherix target enriched multiplex PCR	Respiratory secretions: sputum, endotracheal aspirate, BAL, mini-BAL, nasal wash, nasopharyngeal swab or aspirate, throat gargle	*Streptococcus pneumoniae* *Haemophilus influenzae* *Moraxella catarrhalis* *Staphylococcus aureus* *Methicillin-resistant S. aureus* *Streptococcus pyogenes* *Klebsiella pneumoniae* *Acinetobacter baumanii* *Pseudomonas aeruginosa* *Neisseria meningitidis* *Bordetella pertussis* *Chlamydophila pneumoniae*	Virulence factor: Panton-Valentine leukocidin	1 h	Non-FDA approved Not CE-IVD	[25]
HealthTrackRx Respiratory Tract Infection Plus	Nasopharynx, sputum, Throat	*Acinetobacter baumanii* *Bordetella pertussis* *B. parapertussis* *B. bronchiseptica* *Chlamydia pneumoniae* *Enterobacter aerogenes, cloacae* *Escherichia coli* *Haemophilus influenzae* *Klebsiella pneumoniae, oxytoca* *Legionella pneumophila* *Moraxella catarrhalis* *Mycoplasma pneumoniae* *Proteus mirabilis, vulgaris* *Pseudomonas aeruginosa* *Serratia marcescens* *Staphylococcus aureus* *Streptococcus agalactiae* *Streptococcus pneumoniae* *Streptococcus pyogenes* Mycobacteria: *Mycobacterium avium-intracellulare* *M. kansasii* *Mycobacterium tuberculosis* Fungi: *Aspergillus flavus, fumigatus,* *niger, terreus* *Candida albicans, glabrata,* *parapsilosis, tropicalis* *Candida auris* *Rhizopus* spp., *Mucor* spp. Viruses	Vancomycin resistance: *VanA* *VanB* Macrolide, lincosamide, and streptogramin resistance: *ermB* *ermC* *mefA* Trimethoprim/Sulfamethoxazole resistance: *dfr (A1, A5) sul (1, 2)* Methicillin resistance: *mecA* Fluoroquinolone resistance: *qnrA1* *qnrA2* *qnrB2* Tetracycline resistance: *tet B* *tet M* ESBL: *CTX-M1 (15), M2 (2), M9 (9),* *M8/25* *SHV* Carbapenemase: *IMP* *NDM* *VIM* *OXA-48* *OXA-51* *AmpC* β-lactamase: *ACT* *MIR* *FOX* *ACC*	Within 24 h	Emergency use authorized by the FDA NOT CE-IVD	Health Track Rx, Denton, TX, USA https://www.contractlaboratory.com/labclass/directories/laboratories.cfm?Health-Track-Rx-Inc&i=2D9FBF47ECE72577B2681D3ED27A8979, accessed on 1 November 2021

FDA, Food and Drug Administration; CE-IVD, approved “Conformité Européenne” Marking for in vitro diagnostic medical devices; BAL, broncheo-alveolar lavage.

## Data Availability

Not applicable.
